# Conditions of Mental Enfeeblement

**Published:** 1894-10-27

**Authors:** T. S. Clouston

**Affiliations:** Lecturer on Mental Diseases, Edinburgh University, and Physician Superintendent Royal Asylum, Morningside


### Conditions of Mental Enfeeblement.

By T. S. Clouston, M.D., Lecturer on Mental Diseases, Edinburgh University, and Physician Superintendent Royal Asylum, Morningside.

In psychiatry the term "mental enfeeblement" is not used to cover every kind of mental weakness, as it is popularly employed. It is used first to denote those states of undeveloped brain and weakened mind that are comprised under idiocy and congenital imbecility which are called *dementia* in the books. Secondly, it covers those cases of permanently weakened brain and mind that are left after attacks of melancholia and mania have run their course, and who have not recovered. Those unrecovered patients mostly sink into a more or less mindless or fatuous condition. They become mentally dead, and remain so. Their judgment, their feelings, their memories, and their wills are weakened. They cannot do their work in the world, and have to be supported by others. They fill and silt up the wards of our pauper asylums. It is one of the social problems of the age. "What is to be done with them?" They are often healthy in body so far as food and sleep and coarse nutrition are concerned. But they are really specimens of humanity damaged in the highest region of all. Their faces express no active mental power. Their movements are comparatively slow, and their whole output of energy, bodily and mental, is lowered. This condition is called dementia. About 30 per cent. of all cases of mania, and 25 per cent. of the cases of melancholia end in such dementia. A demented mother sees her child, and the strongest love in nature is seen to be dead. No drawing towards it roused. A demented man, formerly of pride and talents, is ordered about by his social inferiors, and shows no resentment, while he has no interests left in this world and its excitements and amusements. He is dead to all its keener joys and sorrows. His habits would soon become degraded if not looked after. His temperature is about a degree lower than the normal. His brain cells are visibly "degenerated" when seen under the microscope, being in this way co-related to the enfeeblement of his mental faculties.

Dementia is, indeed, a strange condition. There is scarcely any analogy to the state of the brain which causes dementia in any other disease or any other organ. It may be perfectly stationary for fifty years, and is consistent with life and with a good deal of what would be called "health" in a lower sense. Dements suffer no worries, and feel no responsibilities, and so frequently outlive all their relations. I have demented patients in this asylum who have no known relations because every one belonging to them has died off. The workers have worn out; the feeble-minded idler has been well cared for in this asylum, his wants supplied, his incipient diseases looked after and arrested, and his life regulated to suit his mental condition. The brain has passed through a veritable storm in the preliminary attack of mania, and its most delicate mechanism has never recovered its tone and power again to evolve normal mind.

The most typical dementia mostly comes on after acute "adolescent" insanity, which is very hereditary. In many of such cases it seems as if nature had only energy enough to produce the organism, and to bring it up to the age of eighteen or twenty-three, and that the evil hereditary influences come strongly in, produce a storm in the brain cortex—the attack of virility—and leave a water-logged and useless mind mechanism for the rest of life. Certain bodily indications in many of these hereditarily-tainted adolescents who become demented show that from the first there have been developmental deficiencies. A curious v-shaped or compressed and deformed upper palate is one of these conditions. Such adolescent dements have this palate in common with idiots, many criminals, and epileptics. It a very curious common landmark. I am in the habit of describing this form of adolescent dementia as a "postponed idiocy."

Dementia varies enormously in degree from the very slightest impairment in the keenness of the normal mental faculties down to complete mindlessness. The conditions of idiocy and congenital imbecility result from arrested development of the brain either *in utero* or in early childhood. Idiocy affects not only the mental faculties, but all the bodily organs and functions. An idiot is generally small, stunted dwarfed, awkward in movement, and ugly. The teeth are few and ill set, and soon decay. Two-thirds of idiots die early of scrofula and consumption, showing that the tissues are ill nourished, and unresisting to causes of disease. Many of them can be trained in suitable institutions, the form of education being different from normal children; their "treatment," in fact, consists of proper training. This educates the stunted mental faculties up to their capacity by simple appeals to the senses, and above all by attention to bodily and muscular movements, to nutrition, and to health generally. The brain cell of an idiot is just as different under the microscope from the cell of a healthy child as its whole body is from the form of a sprightly healthy child.

The subject of "backwardness" in School Board children, of unteachableness by the ordinary methods, is now attracting much attention in London. There can be no doubt that such children are suffering from the same kind of mental arrest as idiots; but, of course, of far less degree. Dr. Warner and a special committee which he got appointed for the investigation of such children in the London Board schools has discovered a large number of slight bodily and nervous deficiencies in such children, such as want of symmetry in head and face, in eyes and ears, twitchings, malnutrition of various organs, changes in the growth of the body, of the hands, and of the legs. All such investigations will in time educate the public and the teachers to realise that there is a body as well as a mind to educate, to develop, and to train in all children, and that education does not consist in the three R's. It will show that mind, morals, educability, power to do work and to earn a livelihood, are dependent on sound brain and sound bodies. That primary lesson in education is still much needed by parents and teachers.

